# Supplementary material for: Efficacy and safety of traditional Chinese medicine nasal irrigation on chronic rhinosinusitis recovery after endoscopic sinus surgery: A protocol for a systematic review and meta-analysis
Source: PLoS One. 2022 Aug 1;17(8):e0272371. doi: 10.1371/journal.pone.0272371 (PMC9342793; doi:10.1371/journal.pone.0272371)
Supplement: Attachment — Submitted filename: Response to Reviewers.docx [file pone.0272371.s002.docx]

Dear reviewers,

Thank you for your comments concerning our manuscript entitled “Efficacy and safety of traditional Chinese medicine nasal irrigation on chronic rhinosinusitis recovery after endoscopic sinus surgery: A protocol for a systematic review and meta-analysis” (PONE-D-22-04435). Those comments are valuable and very helpful for revising and improving our paper. We have read through comments carefully and have made corrections. Appended to this letter is our point-by-point response to your comments. The comments are reproduced and our responses are given directly afterward in a different color. We highly appreciate your time and consideration.

Sincerely,

Qinxiu Zhang

Appendix

Reviewer #1:

1. English editing should be performed.

Response: Thanks for your valuable suggestions, we have made careful modifications to this manuscript. The green strikethrough represents the deleted content, and the one marked in red represents the new content. For example:

Line34-35: Lund-Kennedy score for ~~of~~ nasal endoscopy, Lund-Mackay score for ~~of~~ sinus computed tomography

Line36: extraction of data, and methodological quality

Line65-66：CRS greatly affects ~~has a great impact on~~ the social economy

Line111: Studies have ~~shown~~ revealed that

Line118-121: ~~In 2011, a~~ A systematic review published in 2011 showed the effectiveness and safety of TCM nasal irrigation for the postoperative recovery of chronic sinusitis after ESS ~~on postoperative recovery after ESS of chronic sinusitis~~ [27].

Line121: many related clinical trials and ~~some~~ new efficacy evaluation

Line127: The research protocol has been registered on ~~with~~ the Open Science Framework ~~website~~ (OSF)

Line144-145: After the operation, nasal irrigation ~~treatment is insisted~~ was performed on the basis of ~~basic~~ standard treatment, regardless of sex, age, and race.

Line170-171: ongoing clinical trials can be searched in the Chinese ~~China~~ clinical trial registry

Line192: we shall contact the article's authors

Line198-199: we will discuss ~~and decide~~ the decision with Zhang Qinxiu.

Line218: ~~Or~~Alternatively, we can eliminate

Line220: after excluding ~~a certain~~ specific literature

Line251: purging intense heat and detoxicating ~~detonicating~~

Line258: after the operation

Line260: after the operation without ~~obvious~~ noticeable adverse reactions

Line263: draining the pus

Line277-278: pointed out that clinicians recommend nasal saline irrigation ~~is recommended by clinicians~~ as an adjuvant treatment

Line282: Thus, ~~the objective of this research is~~ this research aims to formulate a study protocol

2. I should change the titile in PLOS ONE

Efficacy and safety of traditional Chinese'' Effectiveness of traditiona..a systematic review.

Response: Thank you very much for your good suggestion; however, once this protocol is publicly registered in OSF, the content cannot be modified, and the title of the article needs to be consistent with the title registered on the OSF platform, so we cannot modify this issue. We hope you could understand.

3. Abstract

we used..not We will use Review Manager 5.3 software

Response: First of all, thank you for your suggestion; however, we haven't used this software yet, it's something we plan to do, so we still use the simple future tense. Considering your later question about the third person, we have rewritten the sentence by removing "we will use " and inserting "will be applied to" between "software" and "data analysis". Adjusted sentence: ~~We will use~~ Review Manager 5.3 software will be applied to ~~for~~ data analysis. (line38-39)

4. Introduction

- Pediatric chronic rhinosinusitis (CRS) is a common disorder that carries significant morbidity. ……please discuss and cite doi:10.1007/s11882-018-0792-8

Response: Thanks for your suggestion, we have cited this article. The added content is marked in red as follows:

line59-62: CRS is a highly heterogeneous disease, and its pathogenesis is related to the chronic obstruction of the ostiomeatal complex caused by anatomical differences, viral and bacterial infections, allergies, genetics and other factors [6].

5. line 63, An interesting systematic review on single-nucleotide polymorphisms and risk-related chronic rhinosinusitis. …… please cite doi:10.1111/coa.13870

Response: Thanks for your suggestion, we have cited this article. The added content is marked in red as follows:

line62-65: The Chinese epidemiological survey data showed that 11.2% of CRS patients had asthma, and 27.3% had airway hyperresponsiveness [7]. A systematic review conducted by Antonino et al. showed that CRS was closely associated with atopy (49.9%), asthma (50.33%), samter triad (4.9%), and eosinophilia (4.28%) [8].

6. line 67, Endoscopic treatment represents the best therapeutic option that the surgeon can offer. ……please discuss and cite doi:10.1007/s00405-021-06724-6

Response: Thanks for your suggestion, we have cited this article. The added content is marked in red as follows:

Line74-76: The endoscopic treatment represents the best therapeutic option that the surgeon can offer for invasiveness and safety, allowing quick post-surgical, less postoperative pain and fewer complications [10].

7. line 72, Nasal saline irrigation (NSI) plays an important role in the treatment of chronic rhinosinusitis (CRS)…… please discuss and cite doi:10.1002/alr.22330

Response: Thanks for your suggestion, we have cited this article. The green strikethrough means to delete this content, and the added content is marked in red as follows.

line85-88: nasal irrigation can also reduce sinus mucosal inflammation, improve mucociliary clearance, and accelerate the recovery of sinus mucosal structure and function [12-15]. ~~make the drug liquid directly contact the lesions, improve the utilization rate of the drug, and accelerate the recovery of the structure and function of the sinus mucosa [8, 9].~~

8. Methods

please adpat pictos framework

Response: Thank you for your suggestion. We have modified the picture.

9. All verbs in the text must be edited in the third person. For now we write '' We can do the research ... and changing it also in the past: a research has been carried out

Response: Thanks for your excellent suggestion, we've made the appropriate changes. The green strikethrough means deleting the corresponding part, and the content marked in red is the new content. An example is as follows:

line38-39: ~~We will use~~ Review Manager 5.3 software will be applied to data analysis.

Line144-145: After the operation, nasal irrigation ~~treatment is insisted~~ was performed on the basis of ~~basic~~ standard treatment, regardless of sex, age, and race.

Line148-149: The experimental group is ~~nasal irrigation~~ irrigated with Chinese medicine, and the control group is ~~no treatment, or nasal rinse~~ irrigated with normal saline alone

Line207: ~~We will apply Review Manager 5.3 for~~ Review Manager 5.3 will be applied to data analysis.

10. Discuss the bias analysis and major tools used.

Response: In the methodological quality assessment section, we discuss the risk of bias analysis of the included studies and the tools used (line 195-197). We will use the risk of bias assessment tool in the Cochrane Handbook for Systematic Reviews of Interventions (version 5.1.0), together with RevMan 5.3 software for the risk of bias assessment plot. At the same time, in lines 200 to 205, we have made some modifications, the green strikethrough indicates the deletion of the relevant content, and the red part is the new content. The modified part is as follows:

Line200-205: Here are ~~six~~ seven bias items: random sequence generation (selection bias), allocation concealment (selection bias), ~~participant blinding,~~ blinding of subjects and researchers (implementation bias), blinding of outcome assessors (measurement bias), incomplete outcome data (follow-up bias), selective reporting (reporting bias), other bias. Each item will be judged as "low risk of bias", "high risk of bias" and "unclear" according to the risk of bias assessment criteria.

11. Discussion

line 199, The inflammatory nasal mucosa therefore loses the function of mucociliary clearance……please discuss and cite doi:10.1007/s00405-022-07267-0

Response: Thanks for your suggestion, we have cited this article. The added content is marked in red as follows:

Line229-232: The mucociliary clearance function, humidification and filtration function of inflammatory nasal mucosa will reduce. Patients may develop inferior turbinate hypertrophy and nasal congestion and do not respond to standard medical therapy [30].

Reviewer #2:

1. First, TCM nasal irrigations are not clearly and accurately defined in a unique composition……intervention must be a precisely defined medication.

Response: Thank you for your suggestions. According to the theory of traditional Chinese medicine, the main pathogenesis of chronic sinusitis is that the damp-heat in the liver and gallbladder penetrate the nose orifice. After endoscopic surgery, the main syndrome of damp-heat in the liver and gallbladder is mixed with syndromes of qi deficiency and blood stasis.

Under the guidance of this theory, although the composition of the traditional Chinese medicine liquid in the intervention measures is not exactly the same, the treatment principle is generally the same, that is, to achieve the effect of clearing away heat and removing dampness, detoxifying and opening the orifices, strengthening the body and strengthening the exterior, promoting blood circulation and removing blood stasis. Previously, our research group conducted a systematic review and meta-analysis on the treatment of diabetic peripheral neuropathy with traditional Chinese medicine foot bath combined with acupoint massage（DOI: [10.1002/dmrr.3218](https://doi.org/10.1002/dmrr.3218) ）. Although the prescriptions of traditional Chinese medicine foot baths are not identical, most of them have the effect of regulating qi and removing blood stasis.

2. Second, possible disadvantages or complications related to the use of TCM irrigations are not mentioned (i.e. infections) but they are paramount when evaluating the indications to the interventions.

Response: Thanks for your suggestion, we have reviewed the relevant literature and added content about the possible side effects of traditional Chinese medicine nasal irrigation. The red part below is the added content.

line 115-118: Chinese medicine nasal irrigation has high safety and no obvious adverse reactions. Before and after treatment, the patient's liver and kidney functions were normal. Only a few patients experienced nasal pain, nasal itching, and headache during nasal irrigation [24-26].

3. Abstract, lines 24-25: do the Authors mean “after endoscopic sinus susrgery (ESS) for chronic rhinosinusitis (CRS)”? please correct if affirmative

Response: Thanks for your suggestion, we have corrected it here. Green strikethrough means deletion of relevant content, and red font means new content. The revised sentence is as follows:

Line24-26: Continuous comprehensive treatment is still needed after endoscopic sinus surgery (ESS) ~~chronic rhinosinusitis (CRS)~~ for chronic rhinosinusitis (CRS) ~~endoscopic sinus surgery (ESS)~~ to promote the recovery of sinus mucosal morphology and function.

4. Introduction, line 65: I would consider to list every single drug category indicated in CRS or in alternative do not list them at all.

Response: Thanks for your suggestion, we have deleted the sentence "~~Drug therapy includes glucocorticoids, macrolides, mucoactive drugs, etc.~~"(line72)

5. Line 74: “has the characteristics of more minor side effects and easy operation” please revise the English form and the overall concept of the sentence

Response: We appreciate your suggestion, and we have made changes here. Green strikethrough means deletion of relevant content, and red font means new content. The revised sentence is as follows:

Line83-84: has the advantage ~~characteristics~~ of fewer side effects ~~more minor side effects and easy operation~~.

6. Citation 8 and 9 have the same title but different Author; reference nr. 9 is not available in the international databases, the nr. 8 is a Chinese language article; please consider international and English language references. The same comment is related also to References nr. 10, 12, 13, 14, 15, 16 (not available in the international literature or non existent / incorrect DOI)

Response: Thanks for your suggestion, we have deleted references nr. 8, 9, 10 and replaced them with international references nr. 1, 2, 3, 4. The corresponding content is modified as follows (the green strikethrough represents the deleted content, and the red one is the newly added content):

Line85-88: nasal irrigation can also reduce sinus mucosal inflammation, improve mucociliary clearance, and accelerate the recovery of sinus mucosal structure and function [12-15]. ~~make the drug liquid directly contact the lesions, improve the utilization rate of the drug, and accelerate the recovery of the structure and function of the sinus mucosa [8, 9].~~

Line93-98: Jiao et al. found that hypertonic 3.0% saline significantly reduced the epithelial mucociliary or barrier function of cultured human nasal epithelial cells. Moreover, it had apparent cytotoxic effects on human nasal epithelial cells [16]. ~~After washing the surgical cavity with 7% hypertonic saline, it is found that the cilia will stop swinging for a short time, and when the saline concentration is as high as 14.4%, the cilia will stop moving permanently [10].~~

However, no suitable international or English literature could be found to replace references nr. 12, 13, 14, 15, 16. Since the literature on the application of Chinese medicine nasal irrigation in chronic sinusitis after nasal endoscopy is too little in international journals, we only added an English literature on the nasal irrigation of Biyuan Tongqiao Granules (line111: Reference 24. doi: 10.1155/2022/2916700.).

Because these five references are in Chinese, there are not available in the international literature.

The DOI of reference nr. 12 can be found on the Wanfang Data Knowledge Service Platform(<https://d.wanfangdata.com.cn/periodical/ChlQZXJpb2RpY2FsQ0hJTmV3UzIwMjIwNDE1Eg9zemd5Z3kyMDE5MDYwNDYaCHU1Z3U2aDds>).

The DOI of references nr. 13, 14, 15, and 16 can be found on the Chinese DOI website (<http://www.chinadoi.cn/portal/index.htm>). Chinese DOI is the earliest DOI registration service in the Chinese field, and it is also the first in Asia. DOI Services. It is jointly managed and operated by the DOI Registration Authority (RA) officially authorized by IDF - China Institute of Science and Technology Information & Beijing Wanfang Data Co., Ltd.

7. Line 80: please replace “has” with “causes”

Response: Thanks a lot for your suggestion, we have replaced "has" with "causes". The revised sentence is as follows:

Line91: Normal saline causes no irritation to the nasal mucosa

8. Line 87: long-term use is not the point, please clarify the use of intranasal topical corticosteroids in the postoperative period

Response: Thank you very much for your suggestion, we have removed the sentence "~~but they are not suitable for long-term use~~" (line100).

We have added relevant content about corticosteroid nasal irrigation as follows：

Line100-107: A meta-analysis of studies by Yoon et al. showed that the beneficial effect of additional steroids in saline irrigation compared with saline irrigation alone was equivocal in terms of endoscopic score and CRS-related quality of life improvement [18]. Soudry et al. revealed that long-term nasal rinse with budesonide is generally safe, but some patients may experience asymptomatic hypothalamic-pituitary-adrenal axis (HPAA) suppression. Simultaneous use of a nasal steroid spray and a pulmonary steroid inhaler increases the risk of HPAA, especially with daily budesonide nasal irrigation [19].

9. In the section Inclusion criteria please state the chronological eligibility criteria of the studies

Response: Thank you for your excellent suggestion. In the inclusion and exclusion criteria section, we have added the time eligibility criteria for studies. The additions have been marked in red as follows:

line136-138: The deadline for the included documents is December 31,2021. The postoperative nasal irrigation time of patients should be at least one month. If it is less than one month, the literature will not be included.

10. Methods, Line 118: it is not clear what “simultaneously” means, consider explaining or removing it.

Response: Thanks for your suggestion, we have removed "simultaneously". The intention was to indicate that in addition to the diagnosis of chronic sinusitis, the patient also underwent functional endoscopic surgery. After your reminder, we feel that "simultaneously" is redundant, so we delete it. The green strikethrough means to remove this word, and the modified sentence is as follows:

Line143-144: have undergone nasal endoscopic sinus surgery ~~simultaneously~~

11. Interventions section: control group should not include no treatment nor irrigations with glucocorticoids or antibiotics, but only saline irrigation in the postoperative time, while interventions should be represented only by saline irrigations with TCM.

Response: Thank you very much for your suggestion, we have removed " no treatment nor irrigations with glucocorticoids or antibiotics ". The green strikethrough represents the removed content, and the revised sentence is as follows:

line148-152: The experimental group is ~~nasal irrigation~~ irrigated with Chinese medicine, and the control group is ~~no treatment, or nasal rinse~~ irrigated with normal saline alone~~, or nasal rinse with normal saline plus antibiotics and (or) glucocorticoids~~. The experimental group is given Chinese medicine combined with normal saline nasal rinse, while the control group is given ~~only~~ normal saline plus antibiotics and (or) glucocorticoid nasal rinse.
